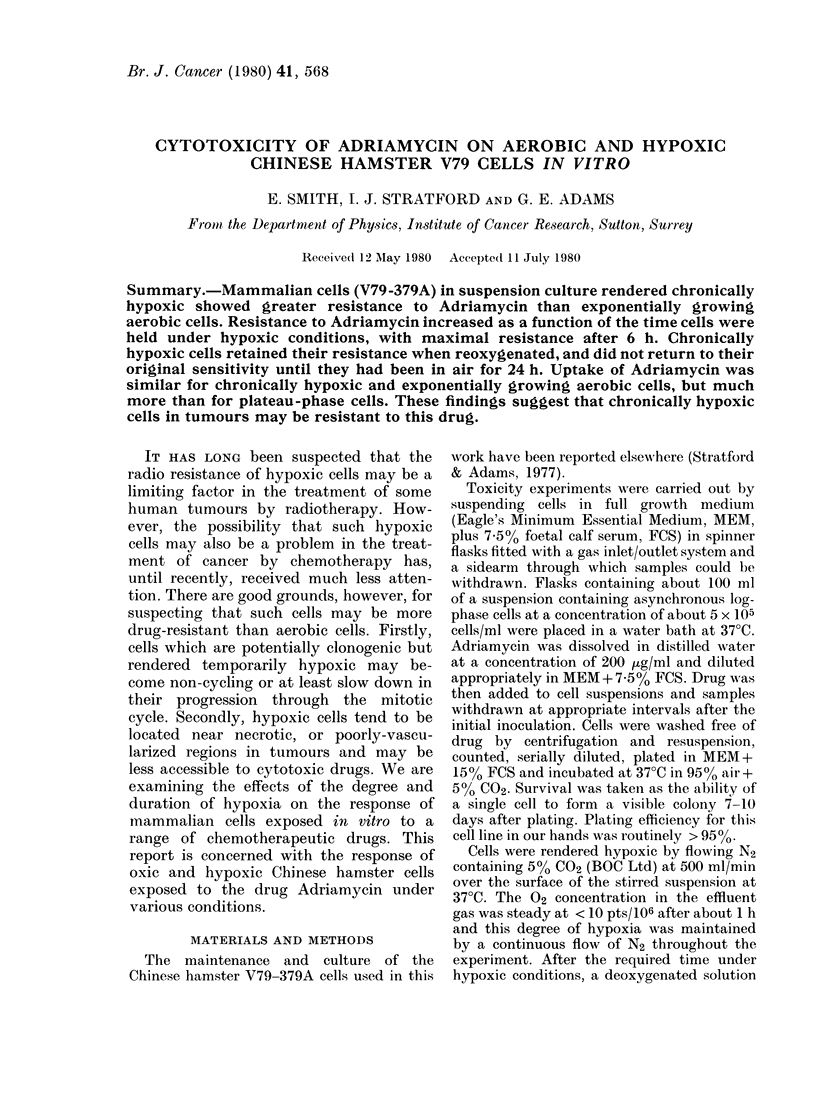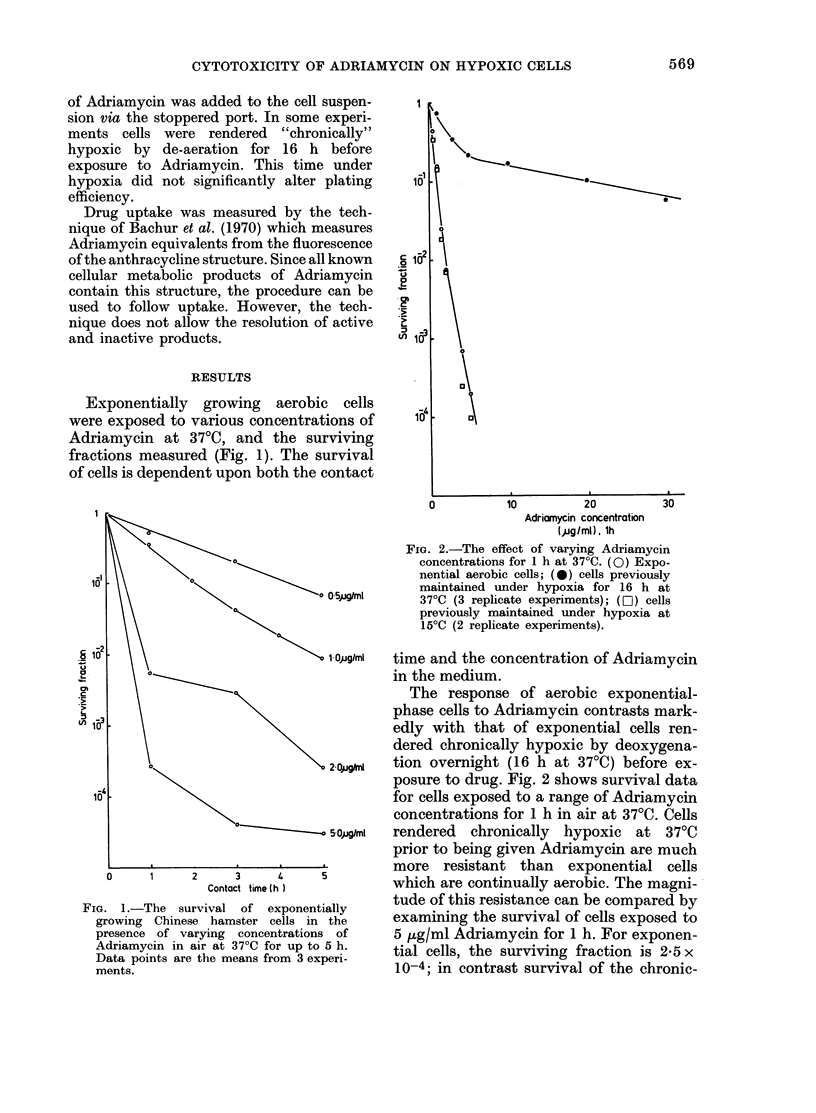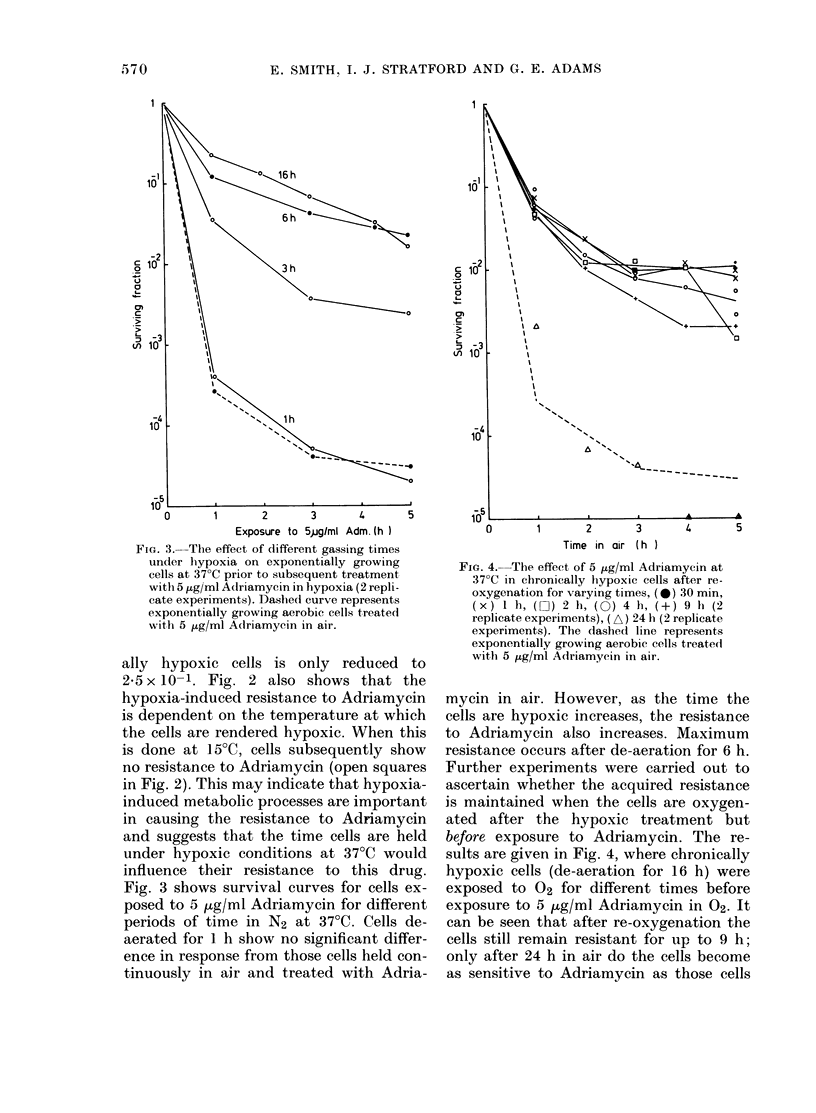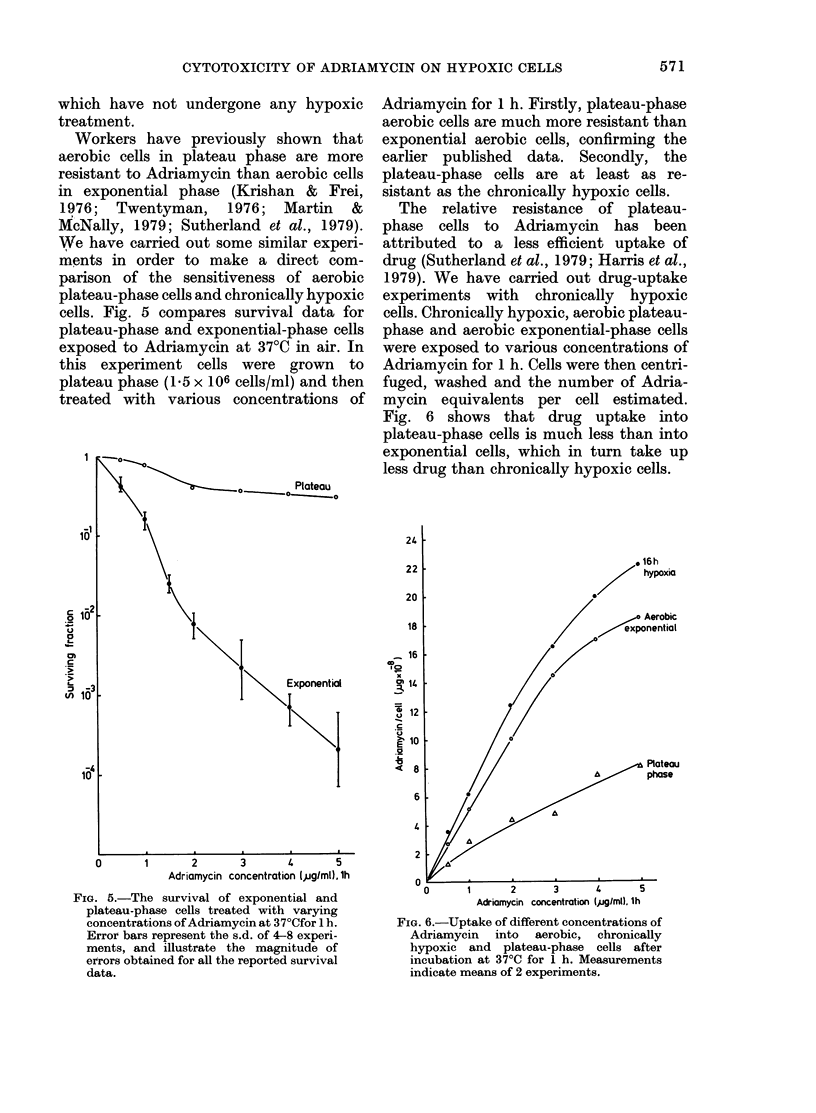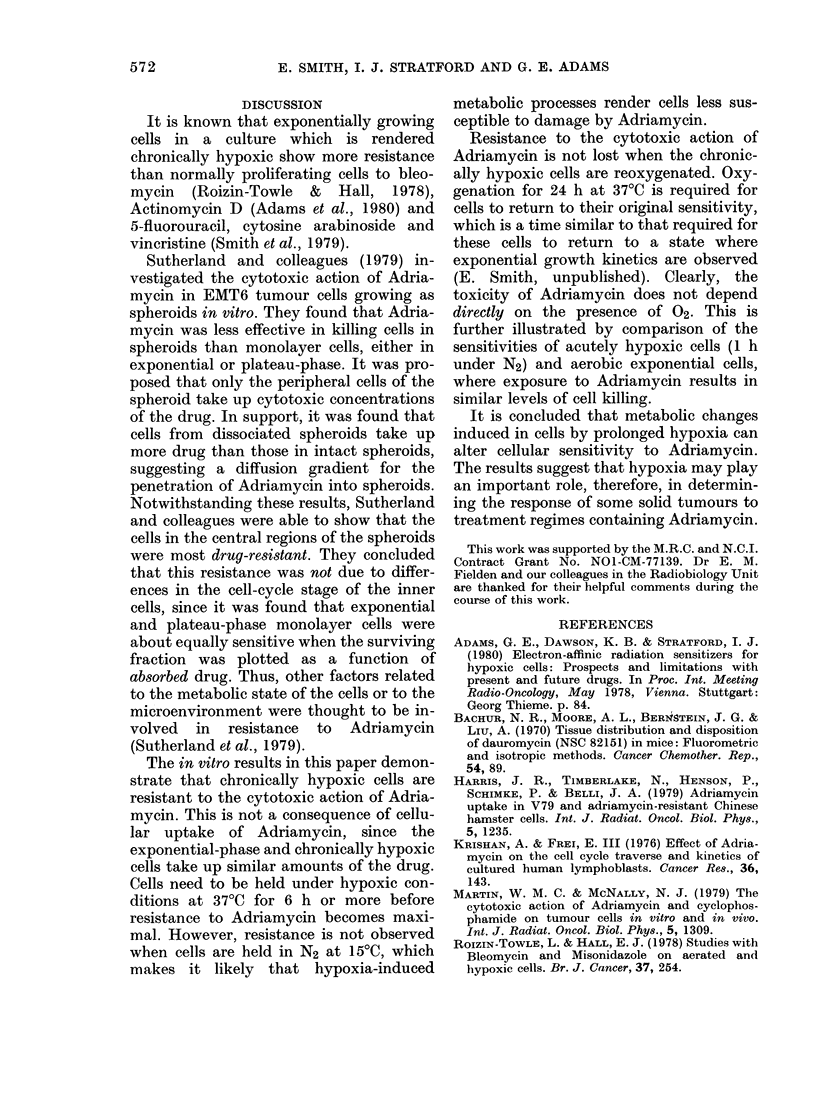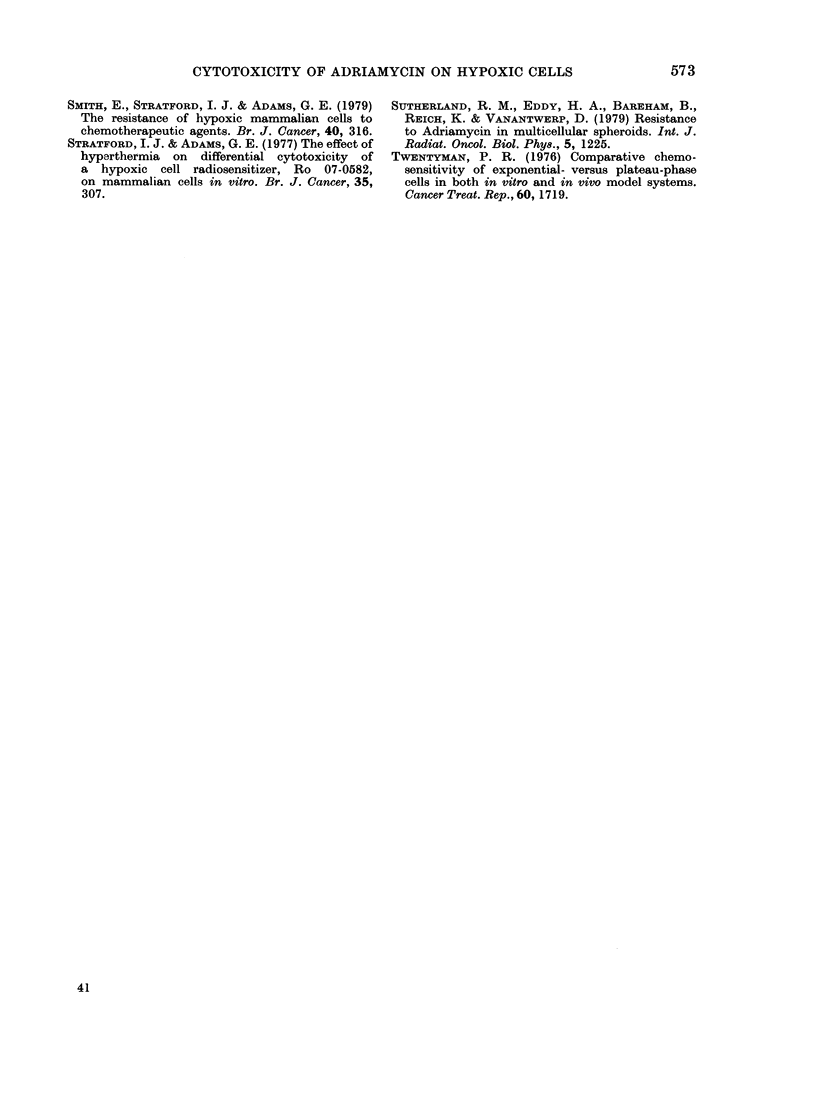# Cytotoxicity of adriamycin on aerobic and hypoxic chinese hamster V79 cells in vitro.

**DOI:** 10.1038/bjc.1980.281

**Published:** 1980-10

**Authors:** E. Smith, I. J. Stratford, G. E. Adams

## Abstract

Mammalian cells (V79-379A) in suspension culture rendered chronically hypoxic showed greater resistance to Adriamycin than exponentially growing aerobic cells. Resistance to Adriamycin increased as a function of the time cells were held under hypoxic conditions, with maximal resistance after 6 h. Chronically hypoxic cells retained their resistance when reoxygenated, and did not return to their original sensitivity until they had been in air for 24 h. Uptake of Adriamycin was similar for chronically hypoxic and exponentially growing aerobic cells, but much more than for plateau-phase cells. These findings suggest that chronically hypoxic cells in tumours may be resistant to this drug.


*Br. J. Cancer* (1980) **41**, 568

### CYTOTOXICITY OF ADRIAMYCIN ON AEROBIC AND HYPOXIC CHINESE HAMSTER V79 CELLS *IN VITRO*

E. SMITH, I. J. STRATFORD AND G. E. ADAMS

*From the Department of Physics, Institute of Cancer Research, Sutton, Surrey*

Received 12 May 1980 Accepted 11 July 1980

**Summary.—Mammalian cells (V79-379A) in suspension culture rendered chronically hypoxic showed greater resistance to Adriamycin than exponentially growing aerobic cells. Resistance to Adriamycin increased as a function of the time cells were held under hypoxic conditions, with maximal resistance after 6 h. Chronically hypoxic cells retained their resistance when reoxygenated, and did not return to their original sensitivity until they had been in air for 24 h. Uptake of Adriamycin was similar for chronically hypoxic and exponentially growing aerobic cells, but much more than for plateau-phase cells. These findings suggest that chronically hypoxic cells in tumours may be resistant to this drug.**

It has long been suspected that the radio resistance of hypoxic cells may be a limiting factor in the treatment of some human tumours by radiotherapy. However, the possibility that such hypoxic cells may also be a problem in the treatment of cancer by chemotherapy has, until recently, received much less attention. There are good grounds, however, for suspecting that such cells may be more drug-resistant than aerobic cells. Firstly, cells which are potentially clonogenic but rendered temporarily hypoxic may become non-cycling or at least slow down in their progression through the mitotic cycle. Secondly, hypoxic cells tend to be located near necrotic, or poorly-vascularized regions in tumours and may be less accessible to cytotoxic drugs. We are examining the effects of the degree and duration of hypoxia on the response of mammalian cells exposed *in vitro* to a range of chemotherapeutic drugs. This report is concerned with the response of oxic and hypoxic Chinese hamster cells exposed to the drug Adriamycin under various conditions.

### MATERIALS AND METHODS

The maintenance and culture of the Chinese hamster V79–379A cells used in this work have been reported elsewhere (Stratford & Adams, 1977).

Toxicity experiments were carried out by suspending cells in full growth medium (Eagle's Minimum Essential Medium, MEM, plus 7·5% foetal calf serum, FCS) in spinner flasks fitted with a gas inlet/outlet system and a sidearm through which samples could be withdrawn. Flasks containing about 100 ml of a suspension containing asynchronous log-phase cells at a concentration of about $5 \times 10^5$ cells/ml were placed in a water bath at 37°C. Adriamycin was dissolved in distilled water at a concentration of 200 $\mu$g/ml and diluted appropriately in MEM + 7·5% FCS. Drug was then added to cell suspensions and samples withdrawn at appropriate intervals after the initial inoculation. Cells were washed free of drug by centrifugation and resuspension, counted, serially diluted, plated in MEM + 15% FCS and incubated at 37°C in 95% air + 5% $CO_2$. Survival was taken as the ability of a single cell to form a visible colony 7–10 days after plating. Plating efficiency for this cell line in our hands was routinely > 95%.

Cells were rendered hypoxic by flowing $N_2$ containing 5% $CO_2$ (BOC Ltd) at 500 ml/min over the surface of the stirred suspension at 37°C. The $O_2$ concentration in the effluent gas was steady at < 10 pts/$10^6$ after about 1 h and this degree of hypoxia was maintained by a continuous flow of $N_2$ throughout the experiment. After the required time under hypoxic conditions, a deoxygenated solution of Adriamycin was added to the cell suspension *via* the stoppered port. In some experiments cells were rendered "chronically" hypoxic by de-aeration for 16 h before exposure to Adriamycin. This time under hypoxia did not significantly alter plating efficiency.

Drug uptake was measured by the technique of Bachur *et al.* (1970) which measures Adriamycin equivalents from the fluorescence of the anthracycline structure. Since all known cellular metabolic products of Adriamycin contain this structure, the procedure can be used to follow uptake. However, the technique does not allow the resolution of active and inactive products.

### RESULTS

Exponentially growing aerobic cells were exposed to various concentrations of Adriamycin at 37°C, and the surviving fractions measured (Fig. 1). The survival of cells is dependent upon both the contact

FIG. 1.—The survival of exponentially growing Chinese hamster cells in the presence of varying concentrations of Adriamycin in air at 37°C for up to 5 h. Data points are the means from 3 experiments.

FIG. 2.—The effect of varying Adriamycin concentrations for 1 h at 37°C. (○) Exponential aerobic cells; (●) cells previously maintained under hypoxia for 16 h at 37°C (3 replicate experiments); (□) cells previously maintained under hypoxia at 15°C (2 replicate experiments).

time and the concentration of Adriamycin in the medium.

The response of aerobic exponential-phase cells to Adriamycin contrasts markedly with that of exponential cells rendered chronically hypoxic by deoxygenation overnight (16 h at 37°C) before exposure to drug. Fig. 2 shows survival data for cells exposed to a range of Adriamycin concentrations for 1 h in air at 37°C. Cells rendered chronically hypoxic at 37°C prior to being given Adriamycin are much more resistant than exponential cells which are continually aerobic. The magnitude of this resistance can be compared by examining the survival of cells exposed to 5 μg/ml Adriamycin for 1 h. For exponential cells, the surviving fraction is $2{\cdot}5 \times 10^{-4}$; in contrast survival of the chronic-

FIG. 3.—The effect of different gassing times under hypoxia on exponentially growing cells at 37°C prior to subsequent treatment with 5 µg/ml Adriamycin in hypoxia (2 replicate experiments). Dashed curve represents exponentially growing aerobic cells treated with 5 µg/ml Adriamycin in air.

FIG. 4.—The effect of 5 µg/ml Adriamycin at 37°C in chronically hypoxic cells after reoxygenation for varying times, (●) 30 min, (×) 1 h, (□) 2 h, (○) 4 h, (+) 9 h (2 replicate experiments), (△) 24 h (2 replicate experiments). The dashed line represents exponentially growing aerobic cells treated with 5 µg/ml Adriamycin in air.

ally hypoxic cells is only reduced to $2{\cdot}5 \times 10^{-1}$. Fig. 2 also shows that the hypoxia-induced resistance to Adriamycin is dependent on the temperature at which the cells are rendered hypoxic. When this is done at 15°C, cells subsequently show no resistance to Adriamycin (open squares in Fig. 2). This may indicate that hypoxia-induced metabolic processes are important in causing the resistance to Adriamycin and suggests that the time cells are held under hypoxic conditions at 37°C would influence their resistance to this drug. Fig. 3 shows survival curves for cells exposed to 5 µg/ml Adriamycin for different periods of time in $N_2$ at 37°C. Cells de-aerated for 1 h show no significant difference in response from those cells held continuously in air and treated with Adriamycin in air. However, as the time the cells are hypoxic increases, the resistance to Adriamycin also increases. Maximum resistance occurs after de-aeration for 6 h. Further experiments were carried out to ascertain whether the acquired resistance is maintained when the cells are oxygenated after the hypoxic treatment but *before* exposure to Adriamycin. The results are given in Fig. 4, where chronically hypoxic cells (de-aeration for 16 h) were exposed to $O_2$ for different times before exposure to 5 µg/ml Adriamycin in $O_2$. It can be seen that after re-oxygenation the cells still remain resistant for up to 9 h; only after 24 h in air do the cells become as sensitive to Adriamycin as those cells which have not undergone any hypoxic treatment.

Workers have previously shown that aerobic cells in plateau phase are more resistant to Adriamycin than aerobic cells in exponential phase (Krishan & Frei, 1976; Twentyman, 1976; Martin & McNally, 1979; Sutherland *et al.*, 1979). We have carried out some similar experiments in order to make a direct comparison of the sensitiveness of aerobic plateau-phase cells and chronically hypoxic cells. Fig. 5 compares survival data for plateau-phase and exponential-phase cells exposed to Adriamycin at 37°C in air. In this experiment cells were grown to plateau phase ($1 \cdot 5 \times 10^6$ cells/ml) and then treated with various concentrations of Adriamycin for 1 h. Firstly, plateau-phase aerobic cells are much more resistant than exponential aerobic cells, confirming the earlier published data. Secondly, the plateau-phase cells are at least as resistant as the chronically hypoxic cells.

The relative resistance of plateau-phase cells to Adriamycin has been attributed to a less efficient uptake of drug (Sutherland *et al.*, 1979; Harris *et al.*, 1979). We have carried out drug-uptake experiments with chronically hypoxic cells. Chronically hypoxic, aerobic plateau-phase and aerobic exponential-phase cells were exposed to various concentrations of Adriamycin for 1 h. Cells were then centrifuged, washed and the number of Adriamycin equivalents per cell estimated. Fig. 6 shows that drug uptake into plateau-phase cells is much less than into exponential cells, which in turn take up less drug than chronically hypoxic cells.

Fig. 5.—The survival of exponential and plateau-phase cells treated with varying concentrations of Adriamycin at 37°Cfor 1 h. Error bars represent the s.d. of 4–8 experiments, and illustrate the magnitude of errors obtained for all the reported survival data.

Fig. 6.—Uptake of different concentrations of Adriamycin into aerobic, chronically hypoxic and plateau-phase cells after incubation at 37°C for 1 h. Measurements indicate means of 2 experiments.

### DISCUSSION

It is known that exponentially growing cells in a culture which is rendered chronically hypoxic show more resistance than normally proliferating cells to bleomycin (Roizin-Towle & Hall, 1978), Actinomycin D (Adams *et al.*, 1980) and 5-fluorouracil, cytosine arabinoside and vincristine (Smith *et al.*, 1979).

Sutherland and colleagues (1979) investigated the cytotoxic action of Adriamycin in EMT6 tumour cells growing as spheroids *in vitro*. They found that Adriamycin was less effective in killing cells in spheroids than monolayer cells, either in exponential or plateau-phase. It was proposed that only the peripheral cells of the spheroid take up cytotoxic concentrations of the drug. In support, it was found that cells from dissociated spheroids take up more drug than those in intact spheroids, suggesting a diffusion gradient for the penetration of Adriamycin into spheroids. Notwithstanding these results, Sutherland and colleagues were able to show that the cells in the central regions of the spheroids were most *drug-resistant*. They concluded that this resistance was *not* due to differences in the cell-cycle stage of the inner cells, since it was found that exponential and plateau-phase monolayer cells were about equally sensitive when the surviving fraction was plotted as a function of *absorbed* drug. Thus, other factors related to the metabolic state of the cells or to the microenvironment were thought to be involved in resistance to Adriamycin (Sutherland *et al.*, 1979).

The *in vitro* results in this paper demonstrate that chronically hypoxic cells are resistant to the cytotoxic action of Adriamycin. This is not a consequence of cellular uptake of Adriamycin, since the exponential-phase and chronically hypoxic cells take up similar amounts of the drug. Cells need to be held under hypoxic conditions at 37°C for 6 h or more before resistance to Adriamycin becomes maximal. However, resistance is not observed when cells are held in $N_2$ at 15°C, which makes it likely that hypoxia-induced metabolic processes render cells less susceptible to damage by Adriamycin.

Resistance to the cytotoxic action of Adriamycin is not lost when the chronically hypoxic cells are reoxygenated. Oxygenation for 24 h at 37°C is required for cells to return to their original sensitivity, which is a time similar to that required for these cells to return to a state where exponential growth kinetics are observed (E. Smith, unpublished). Clearly, the toxicity of Adriamycin does not depend *directly* on the presence of $O_2$. This is further illustrated by comparison of the sensitivities of acutely hypoxic cells (1 h under $N_2$) and aerobic exponential cells, where exposure to Adriamycin results in similar levels of cell killing.

It is concluded that metabolic changes induced in cells by prolonged hypoxia can alter cellular sensitivity to Adriamycin. The results suggest that hypoxia may play an important role, therefore, in determining the response of some solid tumours to treatment regimes containing Adriamycin.

This work was supported by the M.R.C. and N.C.I. Contract Grant No. NO1-CM-77139. Dr E. M. Fielden and our colleagues in the Radiobiology Unit are thanked for their helpful comments during the course of this work.

### REFERENCES

Adams, G. E., Dawson, K. B. & Stratford, I. J. (1980) Electron-affinic radiation sensitizers for hypoxic cells: Prospects and limitations with present and future drugs. In *Proc. Int. Meeting Radio-Oncology, May* 1978, *Vienna*. Stuttgart: Georg Thieme. p. 84.

Bachur, N. R., Moore, A. L., Bernstein, J. G. & Liu, A. (1970) Tissue distribution and disposition of dauromycin (NSC 82151) in mice: Fluorometric and isotropic methods. *Cancer Chemother. Rep.*, **54**, 89.

Harris, J. R., Timberlake, N., Henson, P., Schimke, P. & Belli, J. A. (1979) Adriamycin uptake in V79 and adriamycin-resistant Chinese hamster cells. *Int. J. Radiat. Oncol. Biol. Phys.*, **5**, 1235.

Krishan, A. & Frei, E. III (1976) Effect of Adriamycin on the cell cycle traverse and kinetics of cultured human lymphoblasts. *Cancer Res.*, **36**, 143.

Martin, W. M. C. & McNally, N. J. (1979) The cytotoxic action of Adriamycin and cyclophosphamide on tumour cells *in vitro* and *in vivo*. *Int. J. Radiat. Oncol. Biol. Phys.*, **5**, 1309.

Roizin-Towle, L. & Hall, E. J. (1978) Studies with Bleomycin and Misonidazole on aerated and hypoxic cells. *Br. J. Cancer*, **37**, 254.

SMITH, E., STRATFORD, I. J. & ADAMS, G. E. (1979) The resistance of hypoxic mammalian cells to chemotherapeutic agents. *Br. J. Cancer*, **40**, 316.

STRATFORD, I. J. & ADAMS, G. E. (1977) The effect of hyperthermia on differential cytotoxicity of a hypoxic cell radiosensitizer, Ro 07-0582, on mammalian cells *in vitro*. *Br. J. Cancer*, **35**, 307.

SUTHERLAND, R. M., EDDY, H. A., BAREHAM, B., REICH, K. & VANANTWERP, D. (1979) Resistance to Adriamycin in multicellular spheroids. *Int. J. Radiat. Oncol. Biol. Phys.*, **5**, 1225.

TWENTYMAN, P. R. (1976) Comparative chemosensitivity of exponential- versus plateau-phase cells in both *in vitro* and *in vivo* model systems. *Cancer Treat. Rep.*, **60**, 1719.